# Tinnitus and Mild Hearing Loss From a Kiss

**DOI:** 10.7759/cureus.24955

**Published:** 2022-05-12

**Authors:** Lokesh Goyal, Kunal Ajmera, Sriveer Kaasam, Kavya Koppula

**Affiliations:** 1 Hospital Medicine, CHRISTUS Spohn Hospital Corpus Christi - Shoreline, Corpus Christi, USA; 2 Epidemiology, George Washington University, Reston, USA

**Keywords:** sensorineural hearing loss (snhl), nausea, hearing loss, kiss, management of tinnitus

## Abstract

Hearing loss is a common problem that everyone faces at some point in their life. There are three main types of hearing loss: conductive, sensorineural, and mixed. Conductive hearing loss occurs when an external sound is not able to gain access to the inner ear because of some type of obstruction, e.g., cerumen in the auditory canal and fluid in the middle ear. Sensorineural hearing loss (SNHL) is due to damage to the inner ear, which affects the nerve conduction pathway from the inner ear to the brain. Mixed hearing loss is a combination of both conductive and sensorineural hearing loss. Sensorineural hearing loss (SNHL) is one of the most common types of hearing loss. A kiss on an external auditory canal can create a negative pressure that can affect the bones in the inner ear, therefore causing tinnitus and hearing loss. This case report will discuss sensorineural hearing loss (or sudden sensory neural hearing loss (SSNHL)) caused by an innocent kiss on the patient’s ear.

## Introduction

Sensorineural hearing loss (SNHL) is basically of two types: acquired and congenital. A congenital SNHL is usually genetic, and patients are born with this hearing loss. In some cases, prematurity and infections during pregnancy can be passed from mother to child and cause congenital hearing loss. An acquired SNHL is defined as a hearing loss later in life. Common causes of acquired SNHL include aging, noise, trauma, tumors, and medications. Usually, an acquired SNHL occurs gradually and later in life. Still, in some cases, patients develop sudden acquired SNHL or sudden sensory neural hearing loss (SSNHL), which causes deafness in the unilateral ear, e.g., trauma or an innocent loving kiss [1,2].

## Case presentation

The patient is a 50-year-old female with a past medical history of only hypertension controlled on lisinopril 20 mg daily who presented to the hospital with a chief complaint of severe tinnitus and hearing loss in her right ear. The patient stated that she and her husband were watching a movie at home when her husband suddenly kissed her on the right ear. She stated that suddenly after the kiss, she had an almost complete hearing loss in the right ear along with severe tinnitus and nausea. The patient stated that this happened a few hours ago at home, and her tinnitus and hearing loss do not seem to be getting any better, so she decided to come to the hospital. The patient, in general, is healthy besides her hypertension, which is very well controlled on lisinopril 20 mg daily. She stated that she had a normal hearing before this episode. The patient has never worn any hearing aid. She has never had any significant ear infections in the past. She denied neurological symptoms such as headache, diplopia, vertigo, history of meningioma, cancer, autoimmune disease, vasculitis, history of Lyme disease, fever, or any changes in recent medications. The patient denied diabetes, stroke, recent infection, or any recent travel outside her state or outside the United States. The patient is a software engineer and works from home. The patient had a blood test performed, which was normal, as can be seen in Table 1.

**Table 1:** Blood test

Laboratory tests	Results	Normal ranges
Complete blood count (CBC)	All within normal range	Normal
Comprehensive metabolic panel (CMP)	All within normal range	Normal
Thyroid-stimulating hormone (TSH)	1	0.35-5

**Figure 1:**
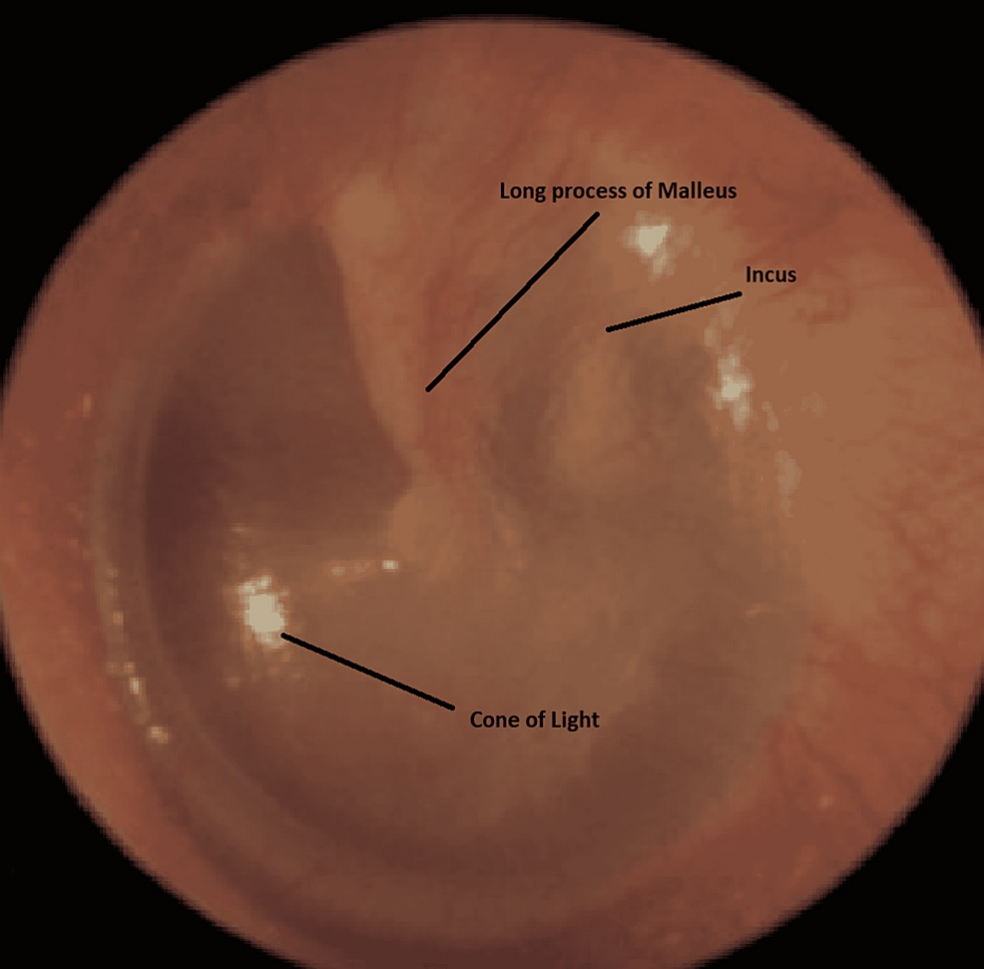
Normal tympanic membrane

On physical examination, the patient had a normal tympanic membrane, as can be seen in Figure 1, with a normal-looking external auditory canal in both ears.

There were no signs of trauma in either ear inside or outside. A full physical examination and skin check were performed, and no signs of physical abuse were found either. Cranial nerves II to XII are all intact. The patient then had CT temporal bone with the brain stem, and an MRI of the brain with contrast was performed as well, but no significant abnormalities were found. In the emergency room, an otolaryngologist was consulted, and the patient was admitted for nausea and severe tinnitus.

The patient was given 4 mg of Zofran IV to help with nausea. The Weber and Rinne tests were performed by the otolaryngologist in the hospital, and the patient was found to have a sensorineural hearing loss only. She was also started on 60 mg prednisone daily by the otolaryngologist. The patient has then discharged from the hospital with a prescription for a tapering dose of prednisone over two weeks and a follow-up appointment with the otolaryngologist outpatient clinic the very next day. The patient had an audiogram performed, which showed a sensorineural hearing loss of 30 decibels (dB) at 500 and 1,000 Hz. The otolaryngologist stated that she had a decrease in stapedial reflex in the right ear, but normal reflex was present in the left ear.

The patient was followed one week later to the outpatient clinic. She stated that she was feeling much better. There was a significant reduction in tinnitus since the incidents, but she can still hear it when she is alone. The patient followed up with ENT again and had the audiological evaluation performed, which showed that she still had sensorineural hearing loss from 15-20 DB at 1,000-2,000 Hz. The patient was advised by ENT to follow up again in two months for reevaluation. The patient stated that she was feeling much better and could work almost normally now.

## Discussion

Sudden sensorineural hearing loss (SSNHL) usually presents as an acute unilateral hearing loss that is sensorineural in nature. Most cases of SSNHL are idiopathic, although it can also occur from multiple other reasons, e.g., infections, cancer, vascular diseases, metabolic, drugs, and trauma.

A kiss on the patient’s external auditory canal creates a large vacuum or negative pressure, which causes an outward pressure on the tympanic membrane. This pressure on the tympanic membrane also creates negative pressure on the small ear bones attached to the tympanic membrane, i.e., stapes, incus, and malleus. This causes dislodging of the stapes in the inner ear, and the fluid in the inner ear (also known as perilymph) leaks out and causes damage to the hair cells, leading to tinnitus and hearing loss [2].

The diagnostic criteria for SSNHL include diagnosing hearing loss as sensorineural, at least 30 dB of hearing loss, and loss of hearing within a 72-hour timeframe [3,4].

Patients who presented with sudden hearing loss should be evaluated within the next few days after symptom onset. The first step is the evaluation of hearing loss itself; this can be achieved using audiometric equipment such as a tone-emitting otoscope or a whisper test. The next step is distinguishing sensorineural hearing loss from conductive hearing loss, which can be achieved by a simple Weber and Rinne test. A focused clinical history and physical examination must be performed on the patient who presents with suspected sudden sensorineural hearing loss (SSNHL), as was in this case. Audiometric testing is necessary to establish the diagnosis of sensorineural hearing loss. Also, an MRI of the brain must be performed with contrast to evaluate for any cochlear pathology [3].

All patients who have SSNHL, including identifiable causes, should be treated with glucocorticoids. For example, patients who have identifiable causes of SSNHL such as meningitis should be treated with antibiotics along with steroids [5]. Treatment with steroids is usually offered for up to 2-8 weeks. We assess the patient’s response to initial therapy with a repeat audiometry test in 2-4 weeks. If the patient experiences greater than 10 dB improvement in hearing, then no further treatment is necessary. However, if there is less than 10 dB improvement, then further treatment needs to be offered, which includes a longer duration of systemic steroids along with possible intratympanic glucocorticoids [6,7].

## Conclusions

A sudden innocent kiss on a person’s external auditory canal can lead to sensorineural hearing loss, which can be devastating and life-altering for the patient. This is also known as Reiter’s ear kiss syndrome first described by Levi Reiter in 2008. Patients such as small children and infants have small and narrow ear canals and are, therefore, at higher risk of this hearing loss. Physicians, medical professionals, parents, and partners should be made aware of the risk associated with this innocent kiss. We should be able to quickly identify and start the treatment as soon as possible once the diagnosis is made to have a better chance of full recovery.
